# Hospital Saturday Fund

**Published:** 1906-01-27

**Authors:** 


### HOSPITAL SATURDAY FUND.

At a meeting of the Hospital Saturday Fund, held at the central offices, Gray's Inn Road, last Saturday, Sir Savile Crossley, Chairman of the Fund, presiding, it was unanimously resolved, on the recommendation of the Distribution Committee, that a sum of £23,631 be awarded to the 202 participating institutions. The total grant was apportioned as follows: £7,429 to 30 general hospitals; £7,086 to 73 special hospitals; £283 to 15 cottage hospitals; £932 to 31 dispensaries; £1,938 to 23 convalescent homes; £300 to the National Sanatoria Association; £389 to 17 nursing institutions; £1,500 to the Surgical Appliance Committee; £120 to the Ambulance Committee, and £2,500 to the Hospital Saturday Fund Distribution Committee.

